# From Erythropoiesis to Circuit Rewiring: Erythropoietin as a Precision Tool for Neurorestoration

**DOI:** 10.3390/ijms27104329

**Published:** 2026-05-13

**Authors:** William Almaguer Melian, Daymara Mercerón Martínez, Briceida Bergado Acosta, Jorge A. Bergado Rosado

**Affiliations:** 1 International Center for Neurological Restoration, Havana 11300, Cuba; william.almaguer@infomed.sld.cu; 2 Centro Interdisciplinario de Neurociencias, University of Valparaiso, Valparaíso 2340000, Chile; daymara.merceron@gmail.com; 3 Centro Interdisciplinario de Neurociencias, Universidad del Sinú “Elías Bechara Zainum”, Carr 1W 38-153, Barrio Juan XXII, Montería 230001, Colombia; briceidabergado@unisinu.edu.co

**Keywords:** erythropoietin, neuroplasticity, synaptic tagging and capture, circuit rewiring, neurorestoration, cognitive recovery

## Abstract

Erythropoietin (EPO), the master regulator of erythropoiesis, is emerging as a pivotal mediator of brain repair. While its capacity to mitigate neural damage is well-documented, we posit that its most profound potential lies in actively orchestrating functional restoration. In the present review we summarize the molecular biology of EPO and the evidence establishing EPO as a potent modulator of neuroplasticity. We use an experimental strategy in which a specific behavioral task marks experience-activated neural circuits, and a subsequent, temporally precise administration of EPO provides a surge of plasticity-related proteins. This creates a synergistic interaction where the proteins are selectively captured by the activated synapses, directing plastic changes with high specificity. We present experimental evidence demonstrating that this synchronized protocol enables the recovery of spatial memory, reinstates synaptic plasticity, and activates genetic programs for plasticity in rodent models of brain injury. Furthermore, we show that endogenous EPO signaling is itself activity-dependent and integral to memory formation. This redefines EPO as a precision tool for neurorestoration, a potential now being pursued with engineered, non-erythropoietic variants of EPO in clinical trials for neurological and psychiatric disorders.

## 1. Introduction

The XXth century witnessed a major transformation in the way we understand the nervous system. In the early years the brain was understood as an immutable organ, able to be damaged but not to recover [1]. However, cumulating evidence during the second half of the century turned the concept; the nervous system is a constantly changing system, both functionally and structurally [2] under the influence of experience.

The experiments demonstrating thicker cortex in animals raised in complex environments [3], the discovery of a relationship between neural function and metabolism [4,5] and that neurogenesis can still take place in the adult brain [6,7] paved the way to a new understanding of the nervous system under the concept of neural plasticity.

Plasticity can take place at different levels, from the molecular to the systemic level, but one of the more relevant is the one modifying synaptic function. Synaptic plasticity can increase the efficacy of synaptic transmission, as in Long-Term-Potentiation [8,9]. Since its discovery LTP has been considered a cellular mechanism of long-term memory [10,11] showing mechanistic coincidences that support this assumption [12,13,14,15]. Synaptic plasticity has also been claimed as a mechanism of recovery after brain lesion [16].

Drugs, neurotransmitters, trophic factors and hormones can modulate synaptic plasticity [17,18]. Erythropoietin (EPO) is a pleiotropic renal hormone that affects not only the production of red blood cells [19] but nerve cell’ function [20,21,22].

The interactions of plasticity influencing factors are time dependent. Studying LTP reinforcement in weakly activated synapses, the existence of a time window for the modulation of previously “tagged” synapses was demonstrated [23,24,25].

The discovery of functional receptors for EPO on neurons [21,26,27] opened an unexpected frontier, prompting investigation into the roles of this classical hormone beyond its hematopoietic domain. A growing body of work from both experimental models and clinical trials has since substantiated that EPO possesses significant neuroprotective and plasticity-promoting effects.

In this review, we will delineate the molecular architecture of EPO signaling, evaluate its role in neuroprotection, and present an expanded focus on its capacity to drive neuroplasticity. We will argue that EPO’s most transformative application lies in its ability to promote the functional rewiring of neural circuits after injury or degeneration. We will synthesize evidence that positions EPO not as a mere shield against damage, but as a precision tool that can be strategically deployed to guide and reinforce the brain’s innate restorative processes.

Our work suggests that functional restoration based on synaptic plasticity may require two timely coordinated signals: first, a behavioral experience that activates synaptic circuits relevant for function, followed by a systemic plasticity-reinforcing factor, such as EPO, delivered within a critical time window. This two-signal strategy offers a powerful new perspective for targeted interventions in restorative neurology and psychiatry.

## 2. Erythropoietin: From Oxygen Sensing to Pleiotropic Signaling

**Figure 1:**
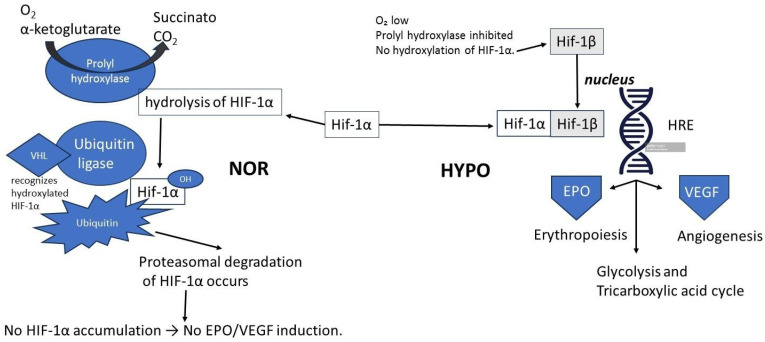
Mechanism of HIF-1α stabilization and EPO induction. Under normoxia (NOR, to the left), HIF-1α is hydroxylated by prolyl hydroxylase (PHD), recognized by the von Hippel–Lindau tumor suppressor protein (VHL), ubiquitinated, and degraded by the proteasome. Under hypoxia (HYPO) hydroxylation is inhibited, allowing HIF-1α to accumulate, dimerize with HIF-1β, and bind to hypoxia-responsive elements (HREs) in the promoter regions of target genes such as EPO (erythropoietin) and VEGF (Vascular endothelial growth factor), promoting erythropoiesis and angiogenesis, respectively.

In 2019, the Swedish Academy announced the awarding of the Nobel Prize in Physiology or Medicine to William G. Kaelin Jr., Sir Peter J. Ratcliffe, and Gregg L. Semenza “for their discoveries of how cells sense and adapt to oxygen availability” (sic: https://www.nobelprize.org/prizes/medicine/2019/press-release/, accessed on 21 October 2019). This is undoubtedly a contribution of enormous significance to understanding a key mechanism in homeostasis. (See Figure 1).

The physiological link between hypoxia, red blood cell production, and humoral factors was first suggested over a century ago by Carnot and Deflandré. Their experiments revealed that plasma from anemic rabbits could stimulate erythropoiesis in healthy recipients, a factor they termed ‘hemopoietin’. This entity was later renamed erythropoietin following the work of Bonsdorf and Jalavisto [28,29].

EPO orchestrates red blood cell production via a classic negative feedback loop [29]: renal EPO release, triggered by tissue hypoxia, stimulates erythropoiesis in the bone marrow to improve oxygen delivery. In recent decades, however, it has become unequivocally clear that EPO is a pleiotropic cytokine with receptors and functions extending far beyond the hematopoietic system, including profound effects on the nervous system. Both animal studies and clinical investigations have supported its potential as a neuroprotective and neurorestorative agent [29,30,31,32,33,34,35,36,37,38,39,40,41,42,43,44,45,46].

While the preponderance of research has focused on EPO’s capacity to prevent neural damage, this review emphasizes a different view. We present an updated synthesis that highlights the molecular biology of EPO and its emerging role in neuroplasticity, the nervous system’s inherent capacity for adaptive structural and functional change in response to experience, learning, or injury. This plasticity-centric view expands EPO’s therapeutic promise from merely preventing damage to actively restoring function after injury or in the context of neurodegeneration.

## 3. Molecular Biology of EPO and Its Central Nervous System Expression

Renal interstitial fibroblasts are the primary source of systemic EPO in response to hypoxia. EPO is a 30 kDa glycoprotein of 165 amino acids [28,29,47], whose circulatory half-life is extended by terminal sialic acid residues that shield it from recognition by galactose receptors on hepatocytes, the cells responsible for its clearance [46]. Its erythropoietic effect stems from an anti-apoptotic action on erythroid progenitor cells expressing the EPO receptor (EPOR). Ligand binding triggers intracellular cascades, beginning with the activation of Janus tyrosine kinase 2 (JAK2), which in turn recruits pathways including MAPK, ERK, and PI3K/Akt, culminating in the upregulation of anti-apoptotic proteins like BCL-XL [48]. This JAK2/STAT5 signaling pathway, upon EPO binding, transmits a potent survival signal to the cell nucleus, inhibiting programmed cell death (apoptosis) and promoting cellular growth and proliferation.

**Figure 2:**
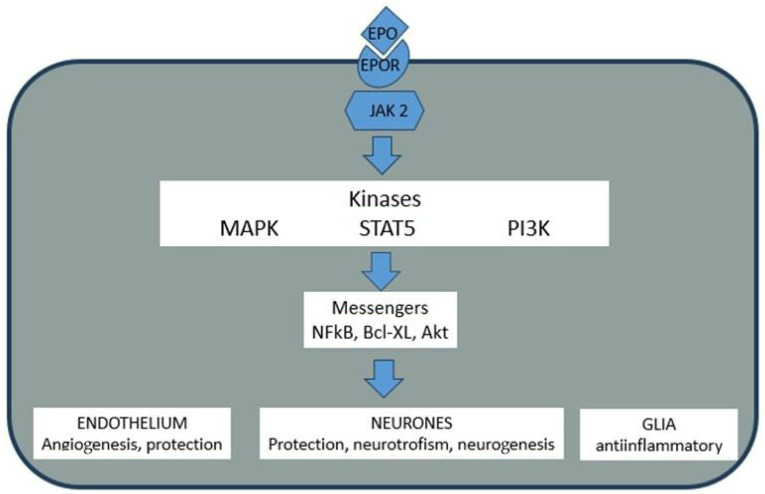
Mechanism of EPO’s effects on sensitive cells bearing the EPO receptor. The molecular cascades activated by EPO-receptor (EPOR) binding have promoting effects on endothelial cells, neurons, and glial cells. EPO: erythropoietin; EPOR: EPO Receptor; JAK2: Janus kinase 2; MAPK: mitogen-activated kinase; STAT5: Transcription activator; PI3K: Phosphatidyl inositol 3 kinase; NFkB: kappaB nuclear factor; Bcl-xL: B cells lymphoma factor; Akt: protein kinase B.

The EPO gene resides on the long arm of chromosome 7 (q11-q22). Its five exons encode a 193-amino acid prohormone, which is proteolytically processed upon secretion to yield the active circulating form. EPO gene transcription is regulated by a complex interplay of factors. A promoter in the 5′ region is activated by GATA-4 and repressed by GATA-2 and contains binding sites for Nuclear Factor-kappa B (NF-κB). The hypoxic induction of EPO is primarily mediated by a Hypoxia Response Element (HRE) in the 3′ region, which serves as a binding site for the Hypoxia-Inducible Factor (HIF) heterodimer (HIF-α/β). The HIF (Hypoxia-Inducible Factor) complex acts as a master regulator of the cellular response to low oxygen, and upon binding to the HRE, it activates the transcription of not only EPO but also a battery of genes involved in angiogenesis, glycolysis, and cell survival, such as Vascular Endothelial Growth Factor (VEGF), glucose transporters, and several glycolytic enzymes [28,32]. See Figure 2.

## 4. EPO and Neuroprotection: An Established Yet Limited Frontier

The clinical use of recombinant human EPO (rhEPO) to correct the anemia of chronic kidney failure provided the impetus and tools to explore its extra-hematopoietic effects [49]. Critical discoveries followed: EPO has actions beyond erythropoiesis [29], neurons possess functional EPO receptors [32], and the hormone can cross the blood-brain barrier [33], a highly selective semi-permeable border of endothelial cells that prevents most substances in the blood from entering the brain, which is a critical requirement for any systemically administered neurotherapeutic. Furthermore, intrinsic production of EPO occurs within the brain itself, with neurons and, most prominently, astrocytes expressing the EPO gene, as demonstrated in humans, non-human primates, and rodents [50]. See Figure 2.

These findings catalyzed intense research, revealing in models of cerebral hypoperfusion that EPO attenuates damage and reduces infarct volume [46], while also stimulating axonal sprouting [48]. Comparable neuroprotection has been documented in traumatic brain injury models [51,52], effects attributable, at least in part, to the hormone’s vasogenic action, its ability to promote the formation of new blood vessels (angiogenesis), thereby improving perfusion and oxygen delivery to compromised neural tissue [53,54]. This protective efficacy extends to neonatal models of hypoxia-ischemia [55] and has shown promise in clinical studies of hypoxic–ischemic encephalopathy in newborns [56].

A diverse and compelling body of evidence points to potential applications for EPO in a wide spectrum of disorders, including optic neuropathy [57], Friedreich’s ataxia [58], multiple sclerosis [36], sleep apnea [59], Alzheimer’s dementia [60], sudden unexpected death in epilepsy (SUDEP) [61], affective disorders [62,63], and, in both animal models [64] and patients of Parkinson’s disease [65,66]. Its potential as a neuroprotectant in preterm neonates continues to garner significant attention [67]. For instance, in optic neuropathy models, EPO administration has been shown to reduce retinal ganglion cell loss and improve visual function [57]. In Friedreich’s ataxia, EPO treatment enhances mitochondrial function and reduces oxidative stress in neuronal models [58]. Furthermore, in multiple sclerosis, EPO exhibits both immunomodulatory and neuroprotective effects, reducing demyelination and promoting remyelination in experimental autoimmune encephalomyelitis [36].

Notwithstanding this promising pre-clinical landscape, the translation of EPO into routine clinical practice for neuroprotection faces substantial hurdles. Meta-analyses of existing studies frequently yield inconclusive results [68]. Furthermore, definitive, rigorous clinical trials in critically ill neurological patients are lacking [69] and the administration of EPO to non-anemic individuals carries risks, including significant alterations in blood viscosity and peripheral resistance [70]. In summary, the future of EPO as a neuroprotective agent remains a vigorously open and critical area for both clinical and basic research. A more comprehensive summary can be found in the review by Hemani and Lane [70].

However, the neuroprotective paradigm focused on mitigating acute damage represents a limited frontier. It often overlooks a fundamental challenge: for patients living with established neurological deficits, true recovery hinges on the brain’s capacity for adaptive neuroplasticity to restore lost function. It is here where we propose to move from the passive concept of protection to the active strategy of guided restoration.

## 5. EPO and Neuroplasticity: Introducing a Strategy for Tag-Guided Circuit Rewiring

Our approach fundamentally reorients the application of EPO from a generalized neuroprotectant to a targeted neurorestorative tool. The critical innovation lies not in EPO itself, but in its strategic application within a synchronized protocol directly inspired by the “synaptic tagging and capture” (STC) hypothesis.

**Figure 3:**
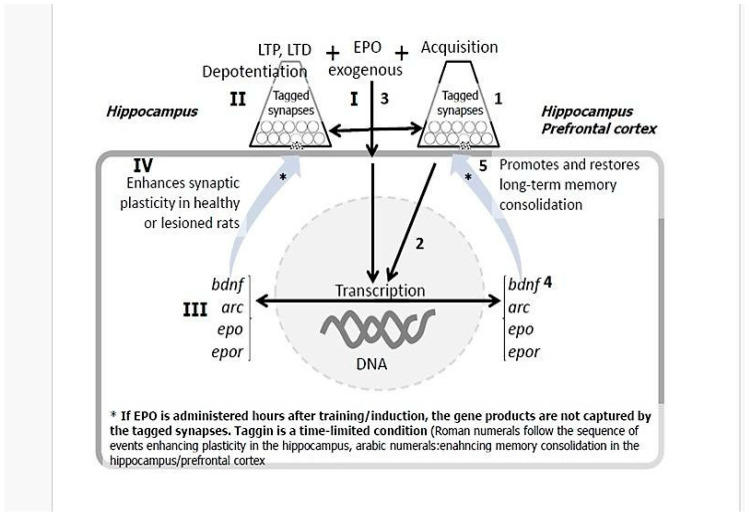
Two-signal strategy for EPO-guided circuit rewiring based on synaptic tagging and capture. On the left, Synaptic Plasticity Axis; on the right, Memory & Behavior Axis. Roman numerals (I–IV) denote processes related to synaptic plasticity modulation. Arabic numerals (1–4) denote processes related to behavioral tagging and memory consolidation. Timely EPO administration (≈10 min post-tagging) enables the capture of plasticity-related proteins by tagged synapses, leading to enhanced plasticity and memory. Delayed administration (5 h) fails to produce these effects. Hippocampus and prefrontal cortex are highlighted as key nodes for synaptic tagging and memory consolidation in this model. *bdnf*: gene for brain-derived growth factor; *arc*: gene for Activity-regulated cytoskeleton-associated protein; *epo*: gene for EPO; *epor*: gene for EPO receptor.

In this protocol, a specific behavioral task (e.g., spatial training) serves as the initial stimulus, tagging activated neural circuits that might be relevant for recovery. The subsequent, temporally precise administration of EPO acts as a powerful facilitator that provides a robust, exogenous surge of plasticity-related proteins (PRPs). This temporally precise coupling ensures that plastic changes are directed with high specificity to the activated circuits, enabling the de novo consolidation of long-term memories and the precise repair of neural pathways that underlie lasting neurological recovery (see Figure 3).

This method moves beyond passive protection to active, circuit-specific rewiring. While the clinical and experimental evidence for EPO’s neuroprotective effect is substantial, the capacity for true functional recovery from established brain damage hinges on neuroplasticity. To directly investigate this capacity, our research group focused on cognitive processes and their cellular substrates, memory and synaptic plasticity, in both healthy animals and models of brain injury.

## 6. EPO Promotes Spatial Memory Recovery and Extends Memory Duration

The synchronized protocol was first tested in a model of established brain damage using a fimbria-fornix (FF) lesion. This tract connects the hippocampus with essential subcortical cholinergic, noradrenergic, and serotonergic inputs, and its injury causes a severe and persistent spatial memory deficit [71,72], modeling aspects of Alzheimer’s dementia [73,74,75]. Crucially, daily administration of EPO 10 min after training in the Morris water maze, a timing aligned with synaptic tagging mechanisms, produced significant recovery of spatial learning in FF-lesioned rats. Retention tests confirmed a stronger memory trace in EPO-treated injured animals. The specificity of this effect is paramount: administration 5 h post-training was ineffective, as was immediate post-injury administration, which would represent a purely neuroprotective strategy [76].

This finding was replicated and extended using the object place recognition test, a low-stress, single-trial spatial memory task. Again, a single EPO dose administered 10 min after acquisition facilitated learning in injured animals and, remarkably, prolonged memory duration from 24 h to 72 h in uninjured animals. The 5 h delay once again abolished the effect [77]. Most recently, we demonstrated that this single, correctly timed EPO dose can prolong memory for at least 21 days [78], suggesting it promotes not only synaptic consolidation but also systemic remote memory, a process where memories, initially dependent on the hippocampus, become stabilized and stored in cortical networks for the long-term [79,80]. The consistent efficacy of the 10 min post-tagging window and the consistent failure of the 5 h delay underscores the temporal precision required for EPO to effectively engage the tagging mechanism.

## 7. EPO Expands the Boundaries of Synaptic Plasticity

Given that learning and recovery are ultimately mediated by changes in synaptic strength, we directly investigated EPO’s effects on synaptic plasticity in the dentate gyrus, focusing on long-term potentiation (LTP) and long-term depression (LTD). LTP and LTD are considered the primary cellular models for information storage in the brain, representing a long-lasting increase or decrease, respectively, in synaptic strength between neurons [8,9,81]. In healthy animals, we found that EPO administration alone induces a slow, progressive potentiation of synaptic transmission [82], akin to the effects of brain-derived neurotrophic factor (BDNF) [83,84,85]. Furthermore, EPO pretreatment lowered the threshold for inducing both LTP (with high-frequency stimulation) and LTD (with low-frequency stimulation) and prevented the reversal of LTP (depotentiation) [82]. This collective evidence indicates that EPO induces a metaplastic state, expanding the dynamic range and stability of synaptic plasticity irrespective of the direction of change, which has profound implications for learning and neurological restoration [82].

The question arises whether EPO could restore this fundamental capacity in a compromised brain. Knowing that FF lesions severely impair synaptic plasticity [86], we demonstrated that a single systemic dose of EPO is sufficient to fully restore long-lasting LTP in the dentate gyrus of lesioned animals [87,88]. As LTP and LTD are established cellular mechanisms of memory [81,89,90,91], this restoration of synaptic malleability provides a plausible substrate for the observed recovery of spatial memory, enabling the formation and stabilization of memory traces (engrams). The finding that EPO facilitates both LTP and LTD suggests it fine-tunes the homeostatic balance of neural networks, enhancing their capacity for information encoding and storage.

## 8. EPO Induces Molecular Mediators of Plasticity in Memory Circuits

The above-mentioned effects imply that EPO must engage the molecular machinery of plasticity. Our data confirms that a single EPO dose rapidly upregulates the expression of two key plasticity-related genes, *bdnf* and *arc*, in the prefrontal cortex of intact animals [76]. BDNF (Brain-Derived Neurotrophic Factor) is a key protein that promotes neuronal survival, differentiation, and synaptic strengthening [76], while Arc (Activity-Regulated Cytoskeleton-associated protein) is crucial for the cytoskeletal remodeling that underlies long-term synaptic changes and memory consolidation [76]. In subsequent studies, both the daily post-training EPO regimen in the Morris water maze and the single-dose protocol in object recognition training increased the expression of these genes in the hippocampus and prefrontal cortex following memory retrieval.

The hippocampus and prefrontal cortex form a critical circuit for explicit memory processing [92,93], with well-defined roles in spatial and object recognition memory [92,93]. Given the established functions of BDNF and Arc in memory consolidation [92,93], it is reasonable to posit that their induction by EPO manages the neuroplastic mechanisms supporting memory recovery and prolongation. Independent work has corroborated that EPO increases *bdnf* and *arc* expression [92,93]. The critical time-dependence of our behavioral effects is mirrored molecularly: animals that received EPO 5 h post-training, which showed no memory recovery, similarly failed to show this specific molecular signature. This supports the hypothesis that EPO modulates neural plasticity within a privileged time window after a salient event, a concept aligned with the ‘synaptic tagging and capture’ hypothesis, where EPO could act as a plasticity-related protein that selectively stabilizes synapses “tagged” by prior learning activity [21,22,77,94,95,96].

Notably, the administration of BDNF itself can induce slow, spontaneous potentiation similar to what we observed after EPO [83,84,85,97,98,99], suggesting that BDNF induction might be a key mediator of EPO’s effects. Furthermore, we found that the expression patterns of *bdnf* and *arc* shift between 24 h and 21 days post-training, indicating that EPO may be accelerating and strengthening the natural process of systems consolidation, with immediate support in the prefrontal cortex leading to more robust long-term storage in the hippocampus.

## 9. Endogenous EPO Signaling Is an Activity-Dependent Component of Memory Formation

To determine if endogenous EPO is a physiological participant in memory processes, we examined its expression following learning. We found that just 3 min of exploration in a spatial object recognition task, which produces short-term memory, significantly increased expression of the EPO gene and its receptor EPO-r, in the hippocampus, with EPO-r expression also rising in the prefrontal cortex. Extending acquisition to 5 min, which extends memory duration to 24 h, produced a much more robust increase in both gene and receptor expression in both brain regions. This suggests that EPO/EPO-r expression is activity-dependent and tuned to the strength of the memory being formed, a finding consistent with reports that motor learning increases EPO signaling [100] and that higher endogenous EPO levels in humans correlate with superior cognitive performance [100]. This positions the endogenous EPO system as a natural, activity-dependent modulator of plasticity, which our synchronized protocol seeks to amplify with exogenous, timed administration.

## 10. The Promise of EPO in Neuropsychiatry

The exploration of EPO’s role in psychiatric disorders, while less advanced, is gaining momentum. A decade-long retrospective search for EPO and schizophrenia yields little, but pioneering work primarily from the group of Kamila Miskowiak provides evidence for its potential use in mood disorders. Their studies indicate that EPO can ameliorate cognitive deficits in depression and counteract cognitive side-effects of electroconvulsive therapy [45,62,101,102,103,104]. Recent pre-clinical evidence further confirms that carbamylated EPO (a non-erythropoietic variant) is effective in animal models of stress-induced depression and anxiety [105,106]. These findings, though preliminary, open a promising avenue for EPO in treating the cognitive deficits often associated with mood disorders, which are frequently resistant to conventional antidepressants. The synchronized protocol suggests a strategic extension: combining cognitive remediation therapy (as the circuit-specific stimulus) with timed administration of non-erythropoietic EPO variants could potentially enhance the efficacy of treating these stubborn cognitive deficits. For example, in a randomized controlled trial, EPO administration improved cognitive performance and hippocampal volume in patients with treatment-resistant depression [62]. In another study, EPO ameliorated cognitive deficits induced by electroconvulsive therapy in depressive patients [45]. Preclinical models further support its efficacy; carbamylated EPO reduces anxiety- and depression-like behaviors in stressed rodents [105].

## 11. Final Considerations and Conclusions

The collective evidence points toward a transformative potential for EPO in treating acquired and degenerative neurological conditions. The experimental foundation provides a robust mechanistic rationale for its use in neurorestoration. We propose that this synchronized, two-signal strategy, coordinating behavioral activation with timed EPO administration, could evolve into a powerful therapeutic tool, representing a conceptual shift in neurological restoration: from a passive, pharmacological shield against damage to an active process of guiding the brain’s innate repair mechanisms to rebuild function. This approach is grounded in a testable hypothesis: the efficacy of EPO in promoting recovery is not merely dose-dependent but is fundamentally “context-dependent,” requiring the prior establishment of a transient, activity-tagged neural circuit. We hypothesize that EPO’s primary restorative role is to broadly supply plasticity-related proteins (PRPs) that are then selectively captured and utilized to stabilize those synaptic pathways that have been functionally “tagged” by a specific experience, thereby achieving a level of precision in circuit rewiring previously unattainable with standalone pharmacological interventions.

Compared to other plasticity-enhancing interventions, such as BDNF mimetics, transcranial magnetic stimulation, or cognitive rehabilitation alone, EPO offers a unique and complementary mechanism. BDNF is widely recognized as a master regulator of synaptic plasticity, capable of inducing plasticity-related gene expression, strengthening long-term potentiation (LTP), and supporting memory consolidation and functional recovery. Indeed, many of the neuroplastic effects elicited by BDNF are comparable to those we have observed with EPO, including the facilitation of LTP, extension of memory duration, and promotion of circuit reorganization after injury. However, what distinguishes our EPO-based strategy is not merely its neurotrophic potential, but rather its temporal precision within the framework of synaptic tagging and capture. While BDNF and other factors can broadly supply plasticity-related proteins (PRPs), the critical determinant of whether these proteins stabilize specific synapses is the timing of their availability relative to the establishment of synaptic tags. Our protocol explicitly leverages this principle by delivering EPO within a narrow post-tagging window (~10 min), thereby ensuring that the surge of PRPs is captured selectively by behaviorally relevant, tagged circuits. This temporally coordinated, two-signal approach, first tagging via experience, then supplying PRPs via EPO, may thus achieve a level of circuit-specific rewiring that is difficult to attain with standalone BDNF administration or other plasticity-modulating interventions lacking such temporal coupling.

The critical importance of temporal precision in this strategy has profound implications for clinical translation. It is important to clarify that EPO’s neuroprotective and pro-plasticity effects are well-established even outside a narrow time window; however, administering EPO within the critical period (approximately 10 min after a salient behavioral or cognitive event) potentiates and directs these effects with exceptional specificity toward circuits that have just been functionally activated. When EPO is delivered shortly after a targeted rehabilitation task, whether motor, cognitive, or sensory, the ensuing surge of PRPs is preferentially captured by synapses ‘tagged’ during that activity. Consequently, the intervention shifts from broad, system-wide modulation to circuit-selective reinforcement, promoting more complete and functionally relevant recovery of the engaged neural pathways.

This paradigm directly informs clinical trial design and rehabilitation protocols. Future trials in chronic neurodegenerative or psychiatric conditions should consider integrating timed EPO administration with structured behavioral interventions, for example, administering intranasal EPO approximately 10 min after a session of cognitive remediation, physiotherapy, or exposure-based therapy. Such an approach would align the pharmacological boost with the neural tagging induced by the therapeutic activity, potentially enhancing the durability and specificity of functional gains. Patient stratification could also be refined by assessing individual differences in endogenous EPO responsiveness or neural activation patterns during task performance. Ultimately, this time-sensitive strategy transforms EPO from a general neurorestorative agent into a precision tool for experience-dependent circuit rewiring, offering a novel framework for personalized neurorehabilitation.

However, the clinical translation of conventional EPO remains constrained, primarily due to safety concerns associated with its erythropoietic effects in non-anemic patients. The solution to this impasse lies in biotechnological innovation. Engineered molecular variants, such as asialoEPO (which lacks terminal sialic acid residues), retain neuroprotective and plasticity-promoting properties but undergo rapid clearance, minimizing systemic erythropoietic effects. This molecule, combined with intranasal delivery to bypass the bloodstream and target the CNS directly, forms the basis of a new therapeutic strategy. The Center for Molecular Immunology in Cuba is currently conducting clinical trials with this intranasal formulation, termed NeuroEPO, in patients with Alzheimer’s disease. It is such targeted efforts, leveraging engineered molecules and precise delivery, that will ultimately define EPO’s place in the neurologist’s and psychiatrist’s arsenal.

Limitations and Future Directions. Despite the promising evidence, several challenges remain. Inter-individual variability in endogenous EPO signaling, the long-term safety of repeated EPO dosing in non-anemic patients, and the generalizability of synaptic tagging mechanisms across different brain regions and disease states are critical open questions. Future research should address these limitations through longitudinal clinical studies, multimodal neuroimaging, and region-specific circuit analyses.

In conclusion, EPO has transcended its classical role in erythropoiesis to emerge as a pleiotropic cytokine with profound effects on the nervous system. The pre-clinical evidence, including that presented here, demonstrates that beyond its well-established neuroprotective role, EPO is a potent promoter of neuroplasticity. It facilitates memory consolidation, expands the capacity of synapses for both potentiation and depression, and activates pro-plasticity genetic programs in hippocampus-cortical circuits. These mechanisms underpin its ability to restore function after established injury, positioning EPO as a unique agent in restorative neurology. Biotechnological developments like nasally administered NeuroEPO, designed to minimize systemic effects, are paving the way for definitive clinical trials to validate this therapeutic potential in neurodegenerative diseases, brain injury sequelae, and neuropsychiatric disorders. By shifting the paradigm from broad-spectrum neuroprotection to timely precise, circuit-specific rewiring, EPO and its engineered derivatives stand poised to redefine therapeutic strategies in restorative neurology and psychiatry.

## Data Availability

No new data were created or analyzed in this study.
